# Morphometric MRI alterations and postoperative seizure control in refractory temporal lobe epilepsy

**DOI:** 10.1002/hbm.22722

**Published:** 2015-02-19

**Authors:** Simon S. Keller, Mark P. Richardson, Jonathan O'Muircheartaigh, Jan‐Christoph Schoene‐Bake, Christian Elger, Bernd Weber

**Affiliations:** ^1^ Department of Molecular and Clinical Pharmacology Institute of Translational Medicine, University of Liverpool Liverpool United Kingdom; ^2^ Department of Radiology The Walton Centre NHS Foundation Trust Liverpool United Kingdom; ^3^ Department of Clinical Neuroscience Institute of Psychiatry King's College London London United Kingdom; ^4^ Department of Neuroimaging Institute of Psychiatry King's College London London United Kingdom; ^5^ Department of Epileptology University of Bonn Bonn Germany; ^6^ Department of Neurocognition/Imaging Life&Brain Research Centre Bonn Germany

**Keywords:** epilepsy surgery, outcome, atrophy, prognosis, MRI, morphometry, thalamus

## Abstract

Refractory mesial temporal lobe epilepsy (mTLE) is a debilitating condition potentially amenable to resective surgery. However, between 40 and 50% patients continue to experience postoperative seizures. The development of imaging prognostic markers of postoperative seizure outcome is a crucial objective for epilepsy research. In the present study, we performed analyses of preoperative cortical thickness and subcortical surface shape on MRI in 115 of patients with mTLE and radiologically defined hippocampal sclerosis being considered for surgery, and 80 healthy controls. Patients with excellent (International League Against Epilepsy outcome (ILAE) I) and suboptimal (ILAE II–VI) postoperative outcomes had a comparable distribution of preoperative atrophy across the cortex, basal ganglia, and amygdala. Conventional volumetry of whole hippocampal and extrahippocampal subcortical structures, and of global gray and white matter, could not differentiate between patient outcome groups. However, surface shape analysis revealed localized atrophy of the thalamus bilaterally and of the posterior/lateral hippocampus contralateral to intended resection in patients with persistent postoperative seizures relative to those rendered seizure free. Data uncorrected for multiple comparisons also revealed focal atrophy of the ipsilateral hippocampus posterior to the margins of resection in patients with persistent seizures. This data indicates that persistent postoperative seizures after temporal lobe surgery are related to localized preoperative shape alterations of the thalamus bilaterally and the hippocampus contralateral to intended resection. Imaging techniques that have the potential to unlock prognostic markers of postoperative outcome in individual patients should focus assessment on a bihemispheric thalamohippocampal network in prospective patients with refractory mTLE being considered for temporal lobe surgery. *Hum Brain Mapp 36:1637–1647, 2015*. © **2015 The Authors Human Brain Mapping Published by Wiley Periodicals, Inc.**

## INTRODUCTION

Mesial temporal lobe epilepsy (mTLE) due to hippocampal sclerosis (HS) is the most common and most frequently operated medically intractable epilepsy disorder [Janszky et al., 2005; Wiebe and Jette, 2012]. It is well documented that patients with mTLE have brain abnormalities beyond the hippocampus. Cortical atrophy has been noted in many MRI morphometric studies, preferentially ipsilateral to the side of the presumed epileptogenic zone [Bernhardt et al., 2009; Keller and Roberts, 2008; Mueller et al., 2009]. Abnormalities of subcortical regions, particularly the thalamus and basal ganglia, have also been frequently demonstrated in groups of patients with mTLE using morphometric magnetic resonance (MR) imaging [Barron et al., 2013; Bernhardt et al., 2012; Dreifuss et al., 2001; Keller and Roberts, 2008], MR diffusion tensor imaging (DTI) [Gong et al., 2008; Keller et al., 2014, 2012], MR spectroscopy [Hetherington et al., 2007], and metabolic positron emission tomography (PET) [Bouilleret et al., 2008; Juhasz et al., 1999] techniques. The thalamus and basal ganglia play important roles in controlling seizure activity throughout the brain, regardless of the location of the epileptogenic focus [Dreifuss et al., 2001]. The thalamus also has an important role in the generation and clinical manifestations of temporal lobe seizures, as evidenced by very early mesial temporal—thalamic ictal coupling in stereoelectroencephalography studies [Guye et al., 2006]. Furthermore, the recent trend to understand mTLE as a systems disorder in context of large scale brain networks [Richardson, 2012] will be inherently related to the structure of the thalamus, basal ganglia and cortex given the known dense and widespread cortico‐subcortical circuitry subserving such networks [Herrero et al., 2002]. However, the significance of subcortical and cortical abnormalities for postoperative seizure outcome in refractory mTLE is poorly understood.

Despite that resective surgery will significantly reduce the number of debilitating seizures for the majority of patients, up to 40% of patients with mTLE and HS will continue to experience postoperative seizures at 2‐year follow‐up [Berkovic et al., 1995]. The proportion of patients continuing to experience any seizure related symptom (e.g., aura, nondisabling seizures) is greater, particularly after longer periods after surgery [de Tisi et al., 2011]. Developing preoperative noninvasive imaging prognostic markers of postoperative seizure outcome is an important research objective, particularly as patient counselling and treatment management could be informed by the identification of a prospective marker of persistent, or even worsening, postoperative seizures.

There is inconsistent evidence for the relationship between hippocampal structural alterations on MRI and postoperative outcome in mTLE, with some studies reporting increased hippocampal atrophy in patients with persistent postoperative seizures relative to those rendered seizure free [Keller et al., 2007; Lin et al., 2005], whereas others report no statistically significant difference between outcome groups [Jack et al., 1992; Mueller et al., 2012; Quigg et al., 1997]. To our knowledge, no study has prospectively examined the relationship between subcortical morphology and postoperative outcome in mTLE. There is also limited data on the relationship between cortical atrophy and outcome. One study reported that, in comparisons with controls, patients with persistent postoperative seizures showed evidence of widespread cortical involvement, whereas patients rendered seizure free showed more restricted pattern of cortical atrophy, although no direct comparisons were performed between patient outcome groups [Yasuda et al., 2010]. Another study reported no significant differences in cortical thickness between patient outcome groups in brain regions shown to be significantly different between patients and controls [Bernhardt et al., 2010]. In the present study, we investigated the relationship between preoperative cortical and subcortical structural alterations and postoperative seizure outcome in a large cohort of patients with refractory mTLE and HS. In particular, we applied sophisticated morphometric approaches to investigate regional alterations in cortical thickness and the surface shape of the mesial temporal lobe, thalamus, and basal ganglia in patients relative to healthy controls, and in patients experiencing persistent postoperative seizures relative to those rendered seizure free.

## METHODS

### Participants

We studied 115 patients with refractory mTLE and neuroradiologically defined ipsilateral HS (mean age 39.2 years, SD 13.2, 75 left mTLE, 40 right mTLE) who were being considered for temporal lobe resection at University Hospital Bonn, Germany. As per standardised protocol, each patient had a detailed clinical assessment to ascertain seizure semiology, interictal electroencephalography (EEG), long‐term video EEG monitoring, if clinically necessary additional intracranial electrode recording, MRI (T1‐weighted, T2‐weighted and Fluid Attenuated Inversion Recovery (FLAIR) images), and neuropsychological assessment [Kral et al., 2002]. HS was identified by an expert neuroradiologist with considerable experience of lesion diagnosis in epilepsy, and was defined by hippocampal volume loss and internal structure disruption on T1‐weighted scans, and/or hyperintensities on T2‐weighted and FLAIR images. All patients had seizures of presumed temporal lobe origin and no evidence of a secondary lesion that may have contributed to seizures. Of the 115 patients, 87 underwent standardised amygdalohippocampectomy [Bien et al., 2013], routine diagnostic histopathological assessment of resected hippocampal specimens by an experienced neuropathologist, and standardised postsurgical seizure outcome follow up, which was assessed using the International League Against Epilepsy (ILAE) outcome classification system [Wieser et al., [Link]]. HS was confirmed in all resected specimens. Forty seven patients (54%) were rendered free from all seizures and aura (ILAE I) while 40 patients (46%) continued to experience persistent postoperative seizures (ILAE II‐VI). Table 1 presents a comparison of clinical data between patient outcome groups. We also recruited a sample of 80 age‐ and sex‐matched neurologically and psychiatrically healthy controls (mean age 40.0 yrs, SD 13.2), who were scanned on the same MRI system using the same MR sequences. All patients and controls provided written informed consent and the local ethics committee approved this study.

**Table 1:** Clinical variables according to outcome

Variable	Seizure free (*n* = 47)	Persistent seizures (*n* = 40)	Confirmatory statistics
Female/male	21/26	26/14	*χ* ^2^ = 2.82, *P* = 0.10
Age at MRI	39.4 (12.6)	39.5 (14.3)	*F* = 0.002, *P* = 0.97
Age at onset of mTLE	16.8 (11.8)	15.3 (12.4)	*F* = 0.33, *P* = 0.57
Presurgical duration of mTLE	20.5 (12.9)	26.2 (16.1)	*F* = 2.25, *P* = 0.13
Febrile convulsions	14 (29.8%)	15 (37.5%)	*χ* ^2^ = 0.28, *P* = 0.59
Meningitis	6 (12.8%)	5 (12.5%)	*χ* ^2^ = 0.001, *P* = 0.97
Seizure frequency	6.6 (10.7)	8.1 (14.6)	*F* = 0.31, *P* = 0.58
SGTCS	12 (25.5%)	21 (52.5%)	*χ* ^2^ = 5.58, *P* = 0.02*
Age at surgery	39.6 (12.5)	39.9 (14.3)	*F* = 0.01, *P* = 0.91
Side of surgery (L/R)	30/19	25/13	*χ* ^2^ = 0.05, *P* = 0.83
Follow up	22.8 (8.2)	23.7 (9.8)	*F* = 0.33, *P* = 0.72

Age at MRI, age at onset, duration of mTLE, and age at surgery are years. Follow up is months. Sex, febrile convulsions, meningitis, and side of surgery are number of cases. Seizure frequency is number per month. Abbreviations: SGTCS, secondary generalized tonic‐clonic seizures.

### MRI and Statistical Analysis

All study participants underwent MRI at the Life & Brain Center in Bonn on a 3 Tesla scanner (Magnetom Trio, Siemens, Erlangen, Germany). An eight‐channel head coil was used for signal reception. Morphometric analyses in this study were performed on 3D T1‐weighted MPRAGE images (160 slices, repetition time (TR) = 1300 ms, inversion time (TI) = 650 ms, echo time (TE) = 3.97 ms, resolution 1.0 × 1.0 × 1.0 mm^3^, flip angle 10°).

We analysed differences in regional cortical thickness between participant groups using Freesurfer software (v.5.3.0; http://surfer.nmr.mgh.harvard.edu/). This approach has been previously described in detail [Dale et al., 1999; Fischl and Dale, 2000] and applied in many quantitative MRI studies [Fischl, 2012]. All images were processed using the standard Freesurfer “recon‐all” processing stream, which supplies the surfaces and morphometry data for each subject. Regional cortical thickness measures are expressed as vertices that represent distances between the reconstructed gray‐white matter boundary and pial surface across the cerebral cortex. After cortical reconstruction and registration to standard space, each dataset was smoothed using a circularly symmetric Gausssian kernel across the cortical surface with a full width at half maximum of 10 mm. The analysis of cortical thickness maps was performed using the query design estimate contrast tool incorporated into Freesurfer software. Group differences were investigated by computing a general linear model of cortical thickness at each vertex of the cortical surface. For group comparisons, the model included cortical thickness as a dependant factor, diagnosis (left mTLE/right mTLE or ILAE I/ILAE II‐VI, and controls) as an independent factor, and participant age as a nuisance variable. We also investigated the relationship between clinical variables and cortical thickness in patients using a regression analysis, including cortical thickness as a dependant factor, clinical data (preoperative duration of mTLE, age of onset of mTLE and preoperative frequency of seizures) as independent factors, and, where specified, patient age as a nuisance variable. Results were reported as statistically significant if they survived correction for multiple comparisons (*P* < 0.05, False Discovery Rate).

We used FSL‐integrated registration and segmentation toolbox (FSL‐FIRST) software, version 5.0 (http://fsl.fmrib.ox.ac.uk/fsl/fslwiki/first) for the automated segmentation, volume calculation and surface shape analysis of the subcortical structures of interest: the left and right hippocampus, amygdala, thalamus, and the major structures of the basal ganglia (putamen, caudate, pallidum, and accumbens area). As described in detail elsewhere [Kim et al., 2013; Patenaude et al., 2011], FSL‐FIRST (i) automatically segmented the structures from the MR images (from which each structure's global volume was obtained using FSL statistical utilities), (ii) parameterised volumetric labels (meshes) from the deformable surfaces of structures, and (iii) permitted vertex‐wise shape analyses along the surface of the segmented structures in standard space after registration to Montreal Neurological Institute space. Permutation testing using “randomise” was used to calculate statistics from surface meshes of the segmented structures, which were thresholded at *P* < 0.05, corrected for multiple comparisons. Results from surface analysis were visualized using Freesurfer's Freeview software. To compare global parenchyma volumes between groups, and to determine intracranial volume (ICV) for normalisation of subcortical volumes, each image was brain extracted using FSL's brain extraction tool [Smith, 2002] and segmented into gray matter, white matter and CSF compartments using FSL's Automatic Segmentation Tool (FAST) [Zhang et al., 2001]. ICV was determined by combining the volumes of gray matter, white matter and CSF.

Comparisons were initially made between healthy controls (*n* = 80), patients with left mTLE (*n* = 75) and those with right mTLE (*n* = 40). We subsequently compared cortical and subcortical morphology between patients who were surgically rendered seizure free and patients who continued to experience persistent postoperative seizures. To increase sample size and thus the sensitivity in detecting pathological alterations in one patient postoperative outcome group relative to another, we side‐flipped the images of patients with right mTLE so that all patients could be analysed together and abnormalities treated as ipsilateral and contralateral to seizure onset/intended surgery only when analysing MRI relationships with postoperative outcome, as undertaken previously [Bernhardt et al., 2010; Lin et al., 2005; Yasuda et al., 2010]. For the statistical comparison of whole structure volumes, a univariate ANOVA was used including group (controls, left mTLE and right mTLE; patients with persistent postoperative seizures and patients rendered seizure free) as factor and volumes (hippocampus, amygdala, thalamus, putamen, caudate, pallidum, accumbens area, gray matter, and white matter) as dependent variables. We included a post hoc Bonferroni correction to control for multiple comparisons and determine the direction of volume differences between groups. Pearson's Correlation Coefficients were used to investigate relationships with clinical variables in patients. We used SPSS (IBM SPSS Statistics, Version 21.0, Armonk, NY) for statistical analysis.

## RESULTS

### Cortex

**Figure 1:**
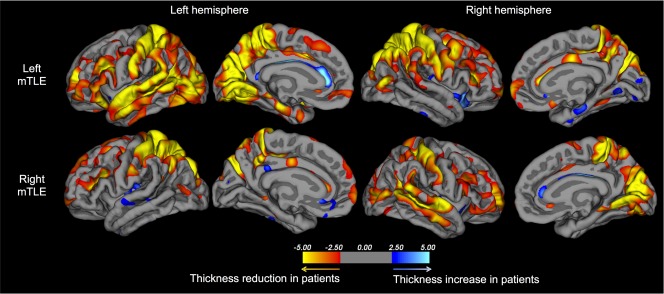
Regional cortical thickness differences between healthy controls and (i) patients with left mTLE (top) and (ii) patients with right mTLE (bottom). Orange‐red coloured regions indicate reduction of cortical thickness in patients relative to controls, and blue‐white coloured regions indicate reduction of cortical thickness in controls relative to patients. The colour bar corresponds to the Z‐score statistic at each vertex.

Patients with left and right mTLE showed evidence of widespread reduction in cortical thickness relative to controls (Fig. 1). In particular, both patient groups had predominantly (or exclusively) ipsilateral reduction of cortical thickness in lateral temporal neocortex (primarily superior and middle temporal gyri), mesial temporal cortex (primarily parahippocampal and entorhinal regions), and temporo‐occipital cortices. Both patient groups also had reduced cortical thickness in bilateral perirolandic areas (predominantly postcentral gyri), mesial parietal cortex including precuneus, lateral parietal cortex, lateral frontal cortex (including all frontal gyri) and cingulate cortex. Some areas of cortical thickness alterations were more extensive in patients with left mTLE, including ipsilateral mesial temporal and temporo‐occipital cortices. There were some circumscribed regions of increased cortical thickness in patients relative to controls, particularly in cingulate regions.

**Figure 2:**
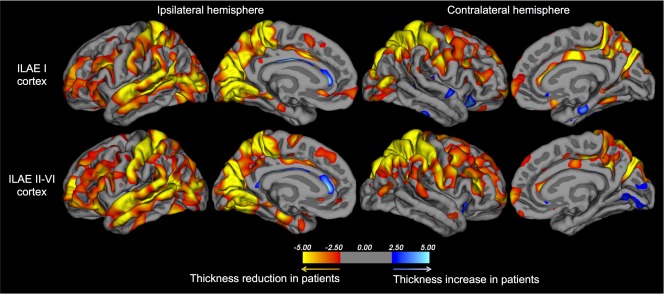
Regional cortical thickness differences between healthy controls and (i) patients rendered seizure free (ILAE I, top) and (ii) patients experiencing persistent postoperative seizures (ILAE II‐VI, bottom). Orange‐red coloured regions indicate reduction of cortical thickness in patients relative to controls, and blue‐white coloured regions indicate reduction of cortical thickness in controls relative to patients. The colour bar corresponds to the *Z*‐score statistic at each vertex.

**Figure 3:**
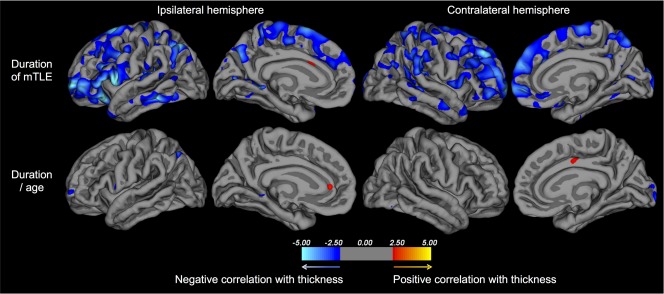
Relationship between preoperative duration of mTLE and cortical thickness across all patients. Duration of mTLE alone is significantly negatively corrected with cortical thickness over widespread multilobar regions (top), as is patient age, which is reflected by the loss of duration‐thickness correlations when age is modelled as a nuisance factor (bottom). The colour bar corresponds to the *Z*‐score statistic at each vertex.

Patients who were rendered seizure free and those who continued to experience postoperative seizures showed very similar patterns of reduced cortical thickness relative to controls (Fig. 2). A reduction in temporal lobe cortical thickness was observed primarily ipsilaterally in both outcome groups, whereas parietal and frontal alterations were bilateral. There were some circumscribed regions of increased cortical thickness in both patients groups relative to controls, again preferentially observed in cingulate cortex. There were no significant differences in regional cortical thickness between patients with persistent seizures and those rendered seizure free when outcome groups were directly compared, even at uncorrected statistical thresholds. Age of onset and preoperative frequency of seizures did not significantly correlate with cortical thickness across all patients. There were significant negative correlations between preoperative duration of mTLE and cortical thickness across multilobar regions, but these findings largely did not survive correction for patient age (Fig. 3). Patient age and preoperative duration of mTLE were significantly correlated (*r* = 0.65, *P* < 0.001).

### Subcortical Structures

Relative to controls, patients with left mTLE had significantly reduced global volume of the left hippocampus (*F* = 119.3, *P* < 0.001), amygdala (*F* = 7.2, *P* = 0.008), thalamus (*F* = 21.4, *P* < 0.001), and putamen (*F* = 6.8, *P* = 0.01), the right amygdala (left mTLE = *F* = 4.1, *P* = 0.05), thalamus (left mTLE = *F* = 7.2, *P* = 0.008), and accumbens area (left mTLE = *F* = 4.7, *P* = 0.03), and whole gray (*F* = 15.2, *P* < 0.001) and white (*F* = 15.2, *P* < 0.001) matter. Significant volume reduction in patients with right mTLE was restricted to the right hippocampus (*F* = 33.3, *P* < 0.001) and accumbens area (*F* = 5.8, *P* = 0.02) relative to controls. Whole structural volumes were not significantly different between patients who were rendered seizure free and those who experienced persistent postoperative seizures (see Supplementary Materials for tabulated results).

**Figure 4:**
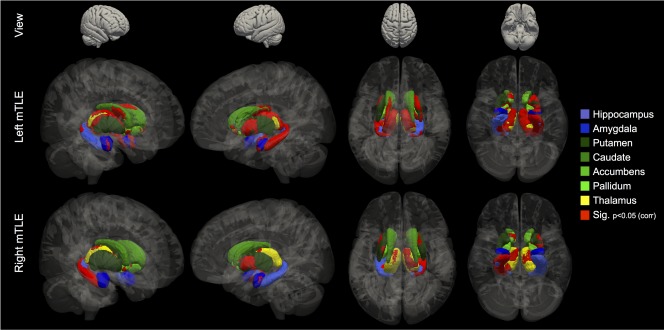
Regional subcortical shape deflation in patients with left and right mTLE relative to controls. Statistically significant vertices corrected for multiple comparisons (red) are projected onto individual subcortical structures.

Results from comparisons of subcortical surface shape between patients with left and right mTLE and controls are presented in Figure 4 (see Supplementary Materials for tabulated results). Patients with left and right mTLE had significant inward surface deflation—a proxy for atrophy—of the ipsilateral hippocampus diffusely, of the amygdala, thalamus, putamen, and pallidum bilaterally, and of smaller focal regions of the contralateral hippocampus. Bilateral thalamic and putamen, and contralateral hippocampal alterations were substantially more widespread in patients with left mTLE. Subtle but significant surface deflation of vertices located in the left and right accumbens was seen in patients with left mTLE, and ipsilaterally in right mTLE. Only patients with left mTLE had alterations of the caudate bilaterally, and patients with right mTLE had a small cluster of vertices inwardly deflating located in the contralateral caudate. There was no regional shape expansion in patients relative to controls.

**Figure 5:**
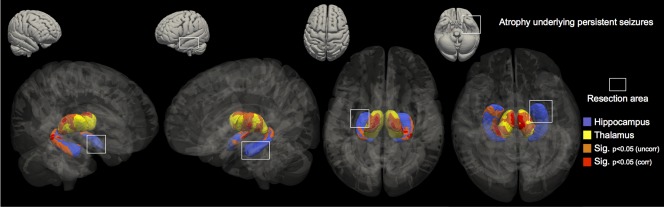
Regional subcortical shape deflation in patients with persistent postoperative seizures relative to patients rendered seizure free. Statistically significant vertices corrected (red) and uncorrected (orange) for multiple comparisons are projected onto the hippocampi and thalami. The white box illustrates the side and approximate area of the hippocampus to be resected. No significant differences between outcome groups in basal ganglia structure were observed.

Patients who continued to experience postoperative seizures had significant regional inward surface deflation of the thalamus bilaterally and of the hippocampus contralateral to the side of intended surgery relative to patients who were rendered seizure free (Fig. 5). Thalamic shape alterations were principally observed in dorsal and ventral regions (ipsilateral cluster sizes = 636, 532, 198, Max *F* = 6.3, Max voxel coordinate = 94, 107, 71; contralateral cluster sizes = 528, 342, 189, Max *F* = 6.1, Max voxel coordinate = 87, 113, 71), and hippocampal alterations in posterior lateral/dorsal areas (cluster size = 872, Max *F* = 7.4, Max voxel coordinate = 70, 95, 63). When we explored results without stringent correction for multiple comparisons, degenerative changes in the posterior ipsilateral hippocampus was observed in patients with persistent postoperative seizures relative to those rendered seizure free (Fig. 5; at *P* < 0.05, uncorrected, cluster size = 746, Max *F* = 4.12, Max voxel coordinate = 113, 92, 70). There were no significant differences in the surface shape of basal ganglia structures or the amygdala between patient outcome groups at corrected or uncorrected statistical thresholds. We furthermore determined the relative shape change for each significant cluster from each comparison in every patient and investigated correlations with clinical variables. There were no significant correlations between age of onset of mTLE or preoperative frequency of seizures and subcortical shape changes (all *P* > 0.05). There was a borderline significant correlation between increasing duration of preoperative mTLE and clusters of maximum shape change in the ipsilateral (*r* = −2.1, *P* = 0.045) and contralateral (*r* = −2.09, *P* = 0.052) thalami across all patients, but not when corrected for patient age (*P* = 0.21 and *P* = 0.36, respectively). No other significant effects were observed.

## DISCUSSION

Refractory mTLE is associated with widespread cortical and subcortical brain atrophy, but it is preoperative focal alterations of the thalamus in both cerebral hemispheres and of the hippocampus contralateral to intended resection that are associated with persistent postoperative seizures. Our patient outcome groups did not differ in cortical thickness, subcortical volume, global gray and white matter, or surface shape of the basal ganglia and amygdala. These findings suggest that persistent postoperative seizures are not simply due to a more widespread distribution of brain damage, but potentially a localized alteration in a bilateral thalamohippocampal network.

### Biological and Clinical Implications

Abnormalities of the thalamus are well established in imaging studies of human mTLE [Barron et al., 2013; Bernhardt et al., 2012; Dreifuss et al., 2001; Gong et al., 2008; Hetherington et al., 2007; Juhasz et al., 1999; Keller et al., 2014, 2012]. In the rodent model of limbic epilepsy, an animal proxy for human mTLE, neuronal loss and physiological alterations of the thalamus have been consistently reported [Bertram and Scott, 2000; Bertram et al., 2001]. Anterior, dorsomedial and pulvinar regions of the thalamus, which have dense bidirectional connections with the temporal lobe, are part of a thalamo‐mesial temporal network structural alteration in patients with mTLE [Keller et al., 2014]. Despite the known presence of thalamic abnormalities in groups of patients with mTLE, this is the first study to reveal a relationship between persistent postoperative seizures and a bihemispheric thalamic structural abnormality. This may suggest that significant thalamic alterations observed in groups of patients with mTLE may be driven by those patients with a subtype of mTLE that will be less responsive to surgical intervention.

Our thalamic structural findings are theoretically compatible with an invasive stereoelectroencephalography study on ictal mesial temporal—thalamic coupling in patients with mTLE [Guye et al., 2006]. This study reported very early involvement of the thalamus during mesial temporal lobe seizures, implicating the thalamus as an important node in an epileptogenic network in mTLE. Importantly, patients with the earliest thalamo‐mesial temporal ictal synchrony had an improved postoperative outcome relative to patients with a later synchrony [Guye et al., 2006]. The authors suggested that a poor postoperative prognosis may be related to an extension of an epileptogenic network to include the thalamus. Based on our anatomical results, it could be argued that the delayed synchrony between the mesial temporal lobe and thalamus in patients with persistent postoperative seizures may relate to impaired thalamotemporal structural connectivity in these patients (if we assume that structural connectivity between two structures shown to be abnormal [hippocampus and thalamus in patients with postoperative seizures] would be impaired). It is important to note that the significance of ictal thalamocortical synchrony as a clinically relevant indicator for epilepsy surgery prognosis has been challenged [Mauguiere, 2007]. Whilst we do not necessarily suggest that thalamic abnormalities are epileptogenic in nature, such impairments may contribute to a less stable functional network which may be more amenable to ictal dynamics [Richardson, 2012].

Some studies that have investigated the volume of the whole hippocampus on MRI have reported no significant association between postoperative outcome and the nonoperated hippocampus in patients with mTLE [Jack et al., 1992; Mueller et al., 2012; Quigg et al., 1997]. Studies using small sample sizes that have used MRI morphometric techniques that permit analysis of regional hippocampal structure have reported that the contralateral hippocampus is increasingly pathologic in patients experience postoperative seizures [Keller et al., 2007; Lin et al., 2005]. Our data are entirely consistent with these five studies, as we observed no relationship between outcome and whole hippocampal volume, but a significant association between outcome and regional atrophy of the posterior lateral and dorsal regions of the contralateral hippocampus. This may suggest that even patients with well‐characterised unilateral seizure onset who are rigorously evaluated for their suitability for temporal lobe surgery, as done in this study, may have bitemporal epileptogenic contributions (if atrophy can be considered a proxy for epileptogenic tissue). A previous study reported that 25% of patients with mTLE and HS who continued to experience seizures had postoperative epileptiform contributions from the contralateral temporal lobe [Hennessy et al., 2000].

We also found evidence of subtle hippocampal atrophy in the posterior ipsilateral hippocampus in patients with postoperative seizures when statistics were uncorrected for multiple comparisons. We reported this finding as preliminary data in an earlier study in a small group of patients with left mTLE [Keller et al., 2007]. Patients with posterior hippocampal seizure onset are more likely to experience persistent postoperative seizures than patients with an anterior mesial temporal lobe onset [Holmes et al., 2000; Prasad et al., 2003], and patients with a poor outcome are more likely to have a distribution of neuronal cell loss throughout the anterior–posterior extent of the hippocampus relative to patients with an excellent outcome who have cell loss confined to anterior regions of the hippocampus [Babb et al., 1984]. Therefore, degeneration of the posterior ipsilateral hippocampus, which is not typically completely resected by amygdalohippocampectomy or anterior temporal lobectomy, may be a marker of a poor postoperative seizure outcome. However, contrary to this suggestion, a previous electrophysiological study indicated that the hippocampal remnant after surgery was the cause of postoperative seizures in only 5% of patients with mTLE and HS [Hennessy et al., 2000]. Moreover, patients with complete resection of mesial temporal lobe structures, including posterior regions, may continue to experience postoperative seizures [Abosch et al., 2002].

Correlations between an increasing duration of mTLE and decreasing brain volume have been used in some studies to infer progressive brain degeneration due to uncontrolled seizures. Ultimately, only longitudinal imaging can resolve whether recurrent seizures cause progressive brain damage, and only when patient brain changes are considered alongside normal maturational changes observed in neurologically healthy people. An increasing duration of mTLE has been related to hippocampal [Bernasconi et al., 2005; Bernhardt et al., 2013; Bonilha et al., 2006], thalamic [Keller et al., 2002; Natsume et al., 2003] and cortical [Bernasconi et al., 2005; Bernhardt et al., 2013; Bonilha et al., 2006; Keller et al., 2002; Kemmotsu et al., 2011] atrophy in some studies. However, we report in the present study that whilst such correlations do exist, they do so in absence of correction for patient age. It is therefore likely that correlations observed between duration of mTLE and brain atrophy as measured from T1‐weighted MRI may be inherently due to normal brain maturation. Conversely, correlations between duration of mTLE and DTI data have been shown to exist even when corrected for patient age [Keller et al., 2014, 2012], and some morphometric studies do reveal age‐corrected correlations [Bonilha et al., 2006].

Our results may provide some insight into the underlying biological mechanisms of seizure severity in mTLE. Consistent with other work [Janszky et al., 2005; McIntosh et al., 2004], we reported that patients with continuing postoperative seizures have a greater preoperative prevalence of SGTCS relative to patients rendered seizure free. Given the importance of the thalamus in primary generalized epilepsies, it is conceivable that thalamic mechanisms play a role in secondary generalization in mTLE. However, previous work has failed to find such a relationship based on conventional volumetry of T1‐weighted MRI [Dreifuss et al., 2001]. We suggest this may be because volumetry of the entire thalamus may obscure subtle pathological changes that can be identified with regional analysis of thalamic morphometry, as discussed below. Animal studies have indicated a potential role of midline thalamic nuclei in secondary generalisation [Bertram et al., 2001; Turski et al., 1984].

### Methodological Issues

It is important to consider that there may be discrete pathological alterations of cortex underlying postoperative seizures that are spatially discordant between patients, and will therefore be obscured in group analyses such as the present study. One electrophysiological study reported that 55% of patients with persistent postoperative seizures were considered to have seizure onset in the ipsilateral temporal neocortex [Hennessy et al., 2000], which will likely include spatially heterogeneous temporal onset zones. Our data indicate that there is no topologically consistent cortical structural alteration or generalized pattern of brain atrophy underlying persistent postoperative seizures. These findings are contrary to a previous report of more generalized brain degeneration being associated with residual seizures [Yasuda et al., 2010]. Equally, the finding of thalamic and contralateral hippocampal degeneration in patients with postoperative seizures must be considered in light of group‐based analyses, and that these structural alterations cannot prospectively unequivocally differentiate between a patient who will develop postoperative seizures and a patient who will not, given natural neuroanatomical variability and relatively low resolution of clinically applicable MRI. However, it is hoped that our novel results can direct other imaging techniques sensitive to brain microstructure, function and metabolism to prospectively focus assessment on circumscribed regions of the thalamus and hippocampus in patients with mTLE who are being considered for surgery. Multimodal imaging approaches may offer the potential to prognosticate individual patients.

The quantitative assessment of subcortical surface shape is a novel way of determining regional atrophy of subcortical structures on MR images. The approach we have used has previously been shown to reveal regional thalamic alterations that are consistent with focal alterations in thalamocortical connectivity [Hughes et al., 2012], and also corroborate the observation of thalamic changes using other morphometric approaches in patients with idiopathic generalized epilepsy [Kim et al., 2013; Saini et al., 2013]. Shape analysis of mesial temporal lobe structures in patients with mTLE has revealed progressive atrophy of subregions of the hippocampus, amygdala and entorhinal cortex [Bernhardt et al., 2013], with increased sensitivity compared to whole structure volumetry [Bernasconi et al., 2005]. Interestingly, the analysis of entorhinal shape in mTLE has indicated higher rates of regional entorhinal atrophy in the contralateral hemisphere in patients who were not rendered seizure free after temporal lobe surgery [Bernhardt et al., 2013]. The analysis of surface shape of subcortical structures is advantageous relative to the analysis of whole structural volumes, given that we found no differences in the latter between patient outcome groups. Only advanced analysis of regional structural morphology provided the means of detecting focal changes associated with persistent postoperative seizures. Conventional whole‐structure volumetry may obscure subtle but legitimate biologically meaningful results [Keller et al., 2014]. Automated segmentation techniques like the ones used in the present study do have significant strengths, such as the avoidance of human measurement error and bias, increased reproducibility of results and widespread applicability of methods. However, there are some potential limitations associated with such techniques, including occasional inaccuracies in structural segmentation, particularly in regions with poor gray‐white matter contrast.

Side‐flipping MRI data in patients with unilateral neurological disorders/lesions is frequently performed in quantitative studies so that data can be pooled and analysed with respect to a primary factor. This was done in the present study, with the primary factor being postoperative outcome, which is an approach taken in previous MRI studies of outcome in mTLE [Bernhardt et al., 2010; Lin et al., 2005; Yasuda et al., 2010], and more frequently in quantitative MRI studies investigating the clinical correlates (e.g. duration of epilepsy) of brain atrophy in mTLE. There is a caveat with this approach specifically when studying patients with mTLE given that previous research has indicated that the side of seizure onset may be an important factor in determining the extent and distribution of brain abnormalities. In particular, previous work has reported that left mTLE is associated with a more diffuse and bilateral distribution of brain abnormalities relative to right mTLE [Ahmadi et al., 2009; Keller et al., 2012; Kemmotsu et al., 2011]. Notwithstanding the larger size group of patients with left mTLE, our data did indicate visually more widespread cortical, and especially subcortical alterations in left mTLE relative to right mTLE. Previous work has demonstrated that the increased number of patients studied with left mTLE does not explain the more diffuse brain alterations seen in these patients relative to smaller groups of patients with right mTLE, as the same differences in brain alterations exist when patients with left mTLE are randomly reduced in number to match those with right mTLE [Keller et al., 2012]. Despite this potential issue, both left and right mTLE groups showed evidence of bilateral thalamic and contralateral hippocampal alterations relative to controls, and our outcome data indicate that the alterations underlying persistent postoperative seizures were located in the thalamus and contralateral hippocampus. We, therefore, envisage that it is the patients who go on to experience postoperative seizures that are driving the thalamic and contralateral hippocampal alterations in *both* patients with left and right mTLE. Finally, it should be noted that the apparent large difference in number of left (*n* = 75) and right (*n* = 40) sided mTLE patients recruited into this study were consecutively recruited. Left mTLE is significantly more prevalent than right mTLE [Janszky et al., 2003], which may be due to a combination of neurodevelopmental and precipitating factors [Keller et al., 2012].

## CONCLUSION

mTLE due to HS is a systems seizure disorder without a circumscribed brain abnormality. There are networked structural alterations in mTLE that support seizure initiation, propagation and modulation, which include the mesial temporal lobe, thalamus, basal ganglia, and cortex. Our data suggest that new imaging techniques that have the potential to unlock prognostic markers of postoperative outcome in individual patients should focus assessment on a bihemispheric thalamohippocampal network in prospective patients with refractory mTLE being considered for temporal lobe surgery. Damage to this network may prognosticate seizure resistance to conventional temporal lobe surgery.

## Supporting information

Supplementary Information
