# Supplementary material for: Morphometric MRI alterations and postoperative seizure control in refractory temporal lobe epilepsy
Source: Hum Brain Mapp. 2015 Feb 19;36(5):1637–47. doi: 10.1002/hbm.22722 (PMC4415572; doi:10.1002/hbm.22722)
Supplement: Supplementary file 1 — Supplementary Information [file HBM-36-1637-s001.docx]

**Morphometric MRI alterations and postoperative seizure control in refractory temporal lobe epilepsy**

Simon S. Keller^1,2,3^

Mark P. Richardson^3^

Jonathan O’Muircheartaigh^4^

Jan-Christoph Schoene-Bake^5,6^

Christian Elger MD^5^

Bernd Weber MD^5,6^

^1^Department of Molecular and Clinical Pharmacology, Institute of Translational Medicine, University of Liverpool, UK

^2^Department of Radiology, The Walton Centre NHS Foundation Trust, Liverpool, UK

^3^Department of Clinical Neuroscience, Institute of Psychiatry, King’s College London, UK

^4^Department of Neuroimaging, Institute of Psychiatry, King’s College London, UK

^5^Department of Epileptology, University of Bonn, Germany

^6^Department of Neurocognition / Imaging, Life&Brain Research Centre, Bonn, Germany

Supplementary Materials

| **Hemisphere** | **Structure** | **F** | **Sig** |
| --- | --- | --- | --- |
| Ipsilateral | Hippocampus | 1.52 | 0.22 |
|  | Amygdala | 0.14 | 0.71 |
|  | Thalamus | 1.77 | 0.19 |
|  | Putamen | 0.23 | 0.63 |
|  | Caudate | 0.10 | 0.75 |
|  | Pallidum | 0.93 | 0.34 |
|  | Accumbens | 1.13 | 0.29 |
| Contralateral | Hippocampus | 1.59 | 0.21 |
|  | Amygdala | 1.46 | 0.29 |
|  | Thalamus | 1.07 | 0.30 |
|  | Putamen | 0.01 | 0.98 |
|  | Caudate | 0.36 | 0.55 |
|  | Pallidum | 0.32 | 0.57 |
|  | Accumbens | 1.14 | 0.29 |
| Whole Grey Matter |  | 1.46 | 0.29 |
| Whole White Matter |  | 0.91 | 0.36 |

Whole-structure volumetric differences between patients rendered seizure free and patients with persistent postoperative seizures.

| **Comparison** | **Structure** | **Cluster size** | **Max F** | **Max x,y,x (vox)** |
| --- | --- | --- | --- | --- |
| **Controls > Left mTLE** | Left Hippocampus | 2130 | 130 | 119, 92, 61 |
|  | Left Amygdala | 702 | 32.1 | 106, 117, 52 |
|  | Left Thalamus | 2446 | 46.9 | 98, 94, 74 |
|  | Left Putamen | 1558 | 38.3 | 114, 117, 81 |
|  | Left Caudate | 1679 | 77.8 | 104, 137, 74 |
|  | Left Pallidum | 497 | 12.3 | 106, 123, 65 |
|  | Left Accumbens | 117 | 9.53 | 99, 145, 67 |
|  | Right Hippocampus | 1517 | 25.2 | 67, 98, 60 |
|  | Right Amygdala | 406 | 11.7 | 62, 127, 46 |
|  | Right Thalamus | 2153 | 20.6 | 83, 105, 85 |
|  | Right Putamen | 1704 | 38.1 | 68, 137, 76 |
|  | Right Caudate | 1353 | 25.1 | 71, 139, 84 |
|  | Right Pallidum | 739 | 21.5 | 75, 131, 74 |
|  | Right Accumbens | 346 | 31.9 | 81, 142, 69 |
|  |  |  |  |  |
| **Controls > Right mTLE** | Left Hippocampus | 403 | 11.3 | 115, 98, 63 |
|  | Left Amygdala | 180 | 14.5 | 107, 116, 53 |
|  |  | 122 | 19.7 | 121, 121, 53 |
|  | Left Thalamus | 1729 | 15.9 | 104, 118, 76 |
|  |  | 110 | 4.67 | 91, 116, 78 |
|  | Left Putamen | 1143 | 27.5 | 114, 116, 78 |
|  |  | 184 | 7.68 | 106, 138, 65 |
|  | Left Caudate | 102 | 4.46 | 104, 137, 75 |
|  | Left Pallidum | 208 | 8.25 | 102, 129, 71 |
|  |  | 177 | 9.15 | 116, 114, 68 |
|  | Left Accumbens | - | - | - |
|  | Right Hippocampus | 1974 | 44.8 | 67, 101, 58 |
|  | Right Amygdala | 525 | 16.6 | 62, 117, 57 |
|  | Right Thalamus | 1677 | 13.1 | 80, 114, 71 |
|  | Right Putamen | 1069 | 18.3 | 67, 133, 80 |
|  | Right Caudate | - | - | - |
|  | Right Pallidum | 355 | 15.8 | 74, 121, 71 |
|  | Right Accumbens | 303 | 20.6 | 81, 143, 69 |

Results from subcortical shape analysis based on comparisons between controls and patients with left mTLE and right mTLE separately. Results pertain to regional surface deflation in patients relative to controls. There was no significant surface expansion in patients relative to controls.
